# Chemical and molecular examinations of some cowpea genotypes using simple sequence repeat and intersimple sequence repeat DNA markers in relation to their cooking quality

**DOI:** 10.1002/fsn3.2400

**Published:** 2021-06-21

**Authors:** Salma A. Soaud, Salama M. Abdel‐Sayyed, Elsayed M. I. Mahgoub, Sameh A. Korma, Ilaria Cacciotti, Abdelmoneim H. Ali, Azhari Siddeeg, Hany A. M. Wafa

**Affiliations:** ^1^ Genetics Department Faculty of Agriculture Zagazig University Zagazig Egypt; ^2^ Department of Food Science Faculty of Agriculture Zagazig University Zagazig Egypt; ^3^ School of Food Science and Engineering South China University of Technology Guangzhou China; ^4^ Department of Engineering INSTM RU University of Rome “Niccolò Cusano” Roma Italy; ^5^ Department of Food Engineering and Technology Faculty of Engineering and Technology Gezira University Wad Medani Sudan

**Keywords:** cooking time, cowpea, ISSR, ranking correlation, SSR

## Abstract

Cowpea is a leguminous crop that has received widespread attention due to its high nutritional value. However, it is prone to losing some of its nutritional content due to the long cooking process. In this study, fourteen cowpea genotypes were evaluated for their chemical properties before and after cooking, along with the effect of different cooking treatments on the cooking time, considered as the main indicator of the cooking quality. Moreover, the correlation between molecular markers (simple sequence repeat (SSR) and intersimple sequence repeat (ISSR)) and the cooking time of cowpea genotypes was determined. The obtained results showed significant differences between all the investigated genotypes before and after the cooking procedure, reflecting significant genotypic and heritability estimates. Kareem 7 and Greenish Black Balady genotypes showed the shortest cooking time. Microwave's treatment manifested the shortest cooking time compared with the other treatments, which appeared as a new approach to improve the cooking quality of cowpea seeds. Spearman's rank correlation showed that the calculated values were smaller than the tabulated value at 0.05, reflecting the existence of a rank correlation between SSR/ISSR‐PCR banding products and the cooking time of cowpea genotypes. Such a study appeared to be a new approach, particularly in Egypt for the proper selection of the optimal cowpea seed on the basis of its cooking quality.

## Graphical abstract

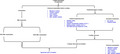

## 1 INTRODUCTION

Cowpea (*Vigna unguiculata* Walp) is a legume crop cultivated in tropical and sub‐tropical countries. This crop exhibits a wide range of differences in its seed characteristics, either morphological or chemical properties (Boukar et al., 2019; Langyintuo et al., 2003). The homeland of cowpea was Southern Africa (Beshir et al., 2019), and later it moved to East and West Africa, and recently India is considered the modern center of diversity of this crop. Cowpea is one of the nutrition staple foods in many parts of the globe (Odedeji & Oyeleke, 2011). Indeed, it is an important source of proteins and carbohydrates already used for the human diet, in addition to minerals, vitamins, and cereals (Gomes et al., 2019; Horn & Shimelis, 2020). Thus, it is a wealthy plant protein source for people who cannot get proteins from animal sources (Akpapunam & Sefa‐Dedeh, 1997), and also for young children as the main component of their diet.

This plant is widely distributed and exhibiting a wide range of variations in its characteristics. Understanding such variabilities seems to be the key to establish some improvement programs. Simple sequence repeat (SSR) and intersimple sequence repeat (ISSR) primers have been widely used for studying genetic varieties among different genotypes. Both markers are advantageous over the others owing to their easier use, lower cost, faster activity, and absence of radioactive substances. SSR and ISSR markers have the potential to identify polymorphisms and determine genetic diversity in intermicrosatellite loci, using primers designed from repetitions of di‐ or tri‐nucleotide simple sequences (Araújo et al., 2019; Badiane et al., 2012; Chen et al., 2017). The polymerase chain reaction (PCR) technique is developed to introduce several assays based on selective DNA amplification, such as SSR and ISSR primers to detect specific variations of nucleotide sequences of polymorphic DNA (Araújo et al., 2019). Moreover, the cowpea seeds are very susceptible to the process treatment and, mainly, to the cooking time, being thermolabile. Therefore, the cooking time is considered the main point of the cowpea seeds cooking quality. Longer cooking time is associated with a decrease in the nutritional value of the cooked seeds. Shorter cooking time is more acceptable and desirable as it would allow reducing the process duration and the energy use, and also saving the associated costs (Hamid et al., 2015; Ngoma et al., 2018).

In this framework, the current study aimed to evaluate fourteen cowpea genotypes for their chemical properties before and after cooking and to investigate the effect of various cooking treatments on the cooking time. Furthermore, the association between SSR or ISSR DNA markers and the cooking time of cowpea genotypes was evaluated since it may play an important role in the selection of the best genotypes for their cooking quality.

## 2 MATERIALS AND METHODS

### 2.1 Cowpea seeds, cultivation conditions, and chemicals

**FIGURE 1:**
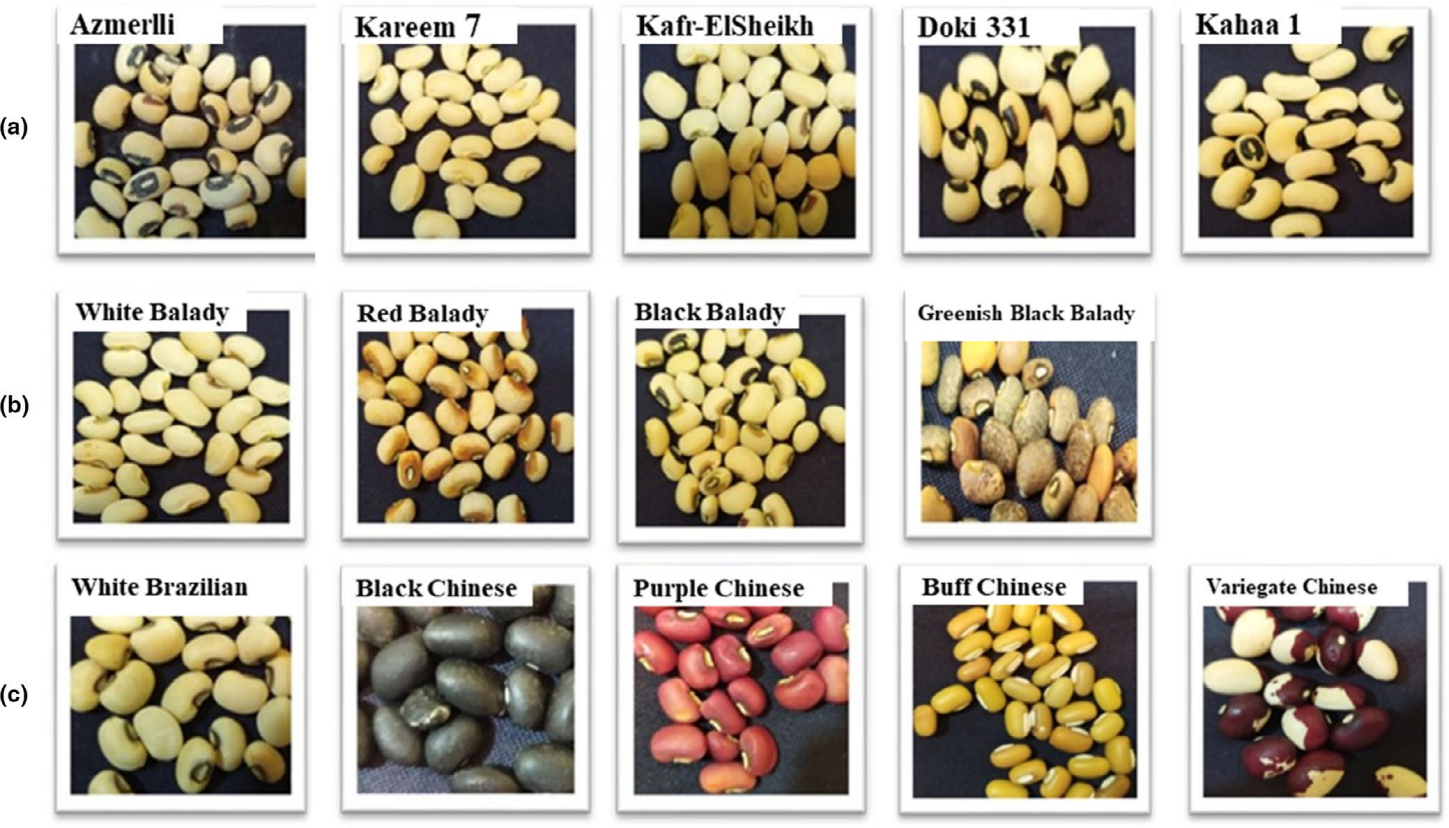
External morphological properties of studied cowpea genotypes; (a) Cultivars genotypes, (b) Balady genotypes, (c) Introduced genotypes

The plant materials used in this study comprised 14 cowpea genotypes, including 9 Egyptian (5 Cultivar and 4 Balady) and 5 introduced ones (1 Brazilian and 4 Chinese) (external morphological properties, Figure 1). The seed of these entries was planted in the summer season of 2018. The plants received equal normal practices of cowpea cultivation to obtain more new seeds. The chemicals and buffer solutions used for molecular analysis such as dNTPs, Tris HCl buffer, Taq DNA polymerase, and Tris‐Borate EDTA buffer were obtained from Sigma‐Aldrich Chemical Co., Ltd. All other reagents and chemicals were of analytical grade, from local suppliers (Egypt).

### 2.2 Chemical examination of cowpea seeds

Five grams of dry cowpea seeds were randomly taken from each genotype and grounded manually. The powdered samples were divided into three replicates. Moisture and protein contents were determined according to Chemists and Horwitz (1975). The ash content was detected by burning the samples in a muffle furnace for 2 hr at 550°C (Hamid et al., 2016). The fibers amount was determined according to the method described by Umar (2014). The nonprotein substances, that is, carbohydrates, lipids, and vitamins were calculated by subtracting protein, ash, and fiber contents from 100.

### 2.3 Cooking technique and treatments

Five grams of each cowpea genotype seed were taken, and three replicates were considered for each kind. The seeds of each genotype/replicate were subjected to different treatments before the cooking procedure. These treatments included airily drying unsoaked seeds, toasting unsoaked seeds, dry heat pretreatment at 60°C for 1 hr, soaking seeds in distilled water for 4 hr, soaking seeds in tap water for 4 hr, soaking seeds in 0.5% of sodium bicarbonate (purity, 99.8%) solution for 4 hr, soaking seeds in 0.5% of baking powder solution for 4 hr, and exposing the soaked seeds in tap water to microwaves for 4 min. A cooking technique was carried out according to Yeung et al. (2009) with major modifications in the cooking procedure. The samples were placed in 250 ml flasks filled with 100 ml of cooking solution or water (tap or distilled) according to the treatments. The flasks were covered with aluminium foils and placed in a boiling water bath. Standard laboratory hotplates were used to maintain uniform and constant temperature during cooking.

Cooking time represents the time from the beginning of the cooking liquid boiling to the complete cooking. During boiling, aliquots of 3–4 seeds were drawn at 5 min intervals up to 20 min, and after 2 min the seeds’ softness was tested by pressing between the index finger and the thumb. When the cooked seeds reached their desirable tenderness, the cooking time was recorded. The optimal time was that corresponded to the softness of 85% of the seeds. At the end of the cooking procedure, each flask's content was transferred to a puncher funnel to separate the cooked seeds from the cooking liquid; the cooked seeds were weighed, put in plastic bags, and kept frozen till examination.

### 2.4 Molecular analysis of cowpea seeds

#### 2.4.1 Genomic DNA extraction

Five seeds from each cowpea entry were germinated in a Petri dish. DNA was extracted from 0.1 g of fresh and young leaves of each entry, according to Dellaporta et al. (1983). The extracted DNA was measured using a UV spectrophotometer (Jenway, 6305) at 280 nm, and its purity was adjusted according to Karp et al. (2012). DNA samples were stored at −20°C till examination by PCR.

#### 2.4.2 SSR and ISSR analysis

SSR and ISSR‐DNA markers technique was used to genetically characterize the studied cowpea genotypes at the molecular level. This technique is based on the amplification of short segments of target genomic DNA using SSR and ISSR primers (Williams et al., 1990). The names and sequences of SSR and ISSR primers used in this study are shown in Table 1. The SSR‐PCR procedure was performed with a final volume of 15 µl, containing 1.5 µl of genomic DNA (50 ng/µl), 1 µl of primer (20 mM), 2.5 µl of dNTPs (2.5 mM), 2 µl of Tris HCl buffer (10 mM, pH 8.3), 0.3 µl of Taq DNA polymerase (three units), 0.5 µl of MgCl_2_ (2 mM), and 7.2 µl of sterile distilled water. While ISSR‐PCR procedure was carried out with a final volume of 10 µl, comprising 1.5 µl of genomic DNA (50 ng/µl), 1 µl of primer (20 mM), 1.3 µl of dNTPs (2.5 mM), 1.5 µl of Tris HCl buffer (10 mM, pH 8.3), 0.2 µl of Taq DNA polymerase (three units), 0.3 µl of MgCl_2_ (2 mM), and 4.2 µl of sterile distilled water. The PCR amplification program was set at 94°C for 4 min (initial denaturation) followed by 35 cycles at 94°C for 90 s, 55°C for 30 s, and 72°C for 90 s, with a final extension at 72°C for 10 min.

**TABLE 1:** Codes and sequence of simple sequence repeat (SSR) and intersimple sequence repeat (ISSR) primers

No.	SSR primers		ISSR primers	
	Codes	Sequence 5′–3′	Codes	Sequence 5′–3′
1	Vm3	5′ GAG CCG GGT TCA ATA GGT A 3′ 5′ GAG CCA GGG CAC AGG TAG T 3′	ISSR825	ACACACACACACACT
2	Vm11	5′ CGG GAA TTA ACG GAG TCA CC 3′ 5′ CCC AGA GGC CGC TAT TAC AC 3′	UBC835	AGAGAGAGAGAGAGAGYC
3	Vm12	5′ TTG TCA GCG AAA TAA GCA GAG A 3′ 5′ CAA CAG ACG CAG CCC AAC T 3′	UBC814	CTCTCTCTCTCTCTCAT
4	Vm13	5′ CAC CCG TGA TTG CTT GTT G 3′ 5′ GTC CCC TCC CTC CCA CTG 3′	UBC826	ACACACACACACACACC
5	Vm14	5′ AAT TCG TGG CAT AGT CAC AAG AGA 3′ 5′ ATA AAG GAG GGCATA GGG AGGTAT 3′	UBC827	ACACACACACACACACG
6	Vm19	5′ TAT TCA TGC GCC GTG ACA CTA 3′ 5′ TCG TGG CAC CCC CTA TC 3′	UBC840	GAGAGAGAGAGAGAGATT
7	Vm22	5′ GCG GGT AGT GTA TAC AAT TTG 3′ 5′ GTA CTG TTC CAT GGA AGA TCT 3′	UBC808	AGAGAGAGAGAGAGAGC
8	Vm23	5′ AGA CAT GTG GGC GCA TCT G 3′ 5′ AGA CGC GTG GTA CCC ATG TT 3′	UBC811	GAGAGAGAGAGAGAGC
9	Vm25	5′ CCA CAA TCA CCG ATG TCC AA 3′ 5′ CAA TTC CAC TGC GGG ACA TAA 3′	UBC868	GAAGAAGAAGAAGAAGAA
10	Vm26	5′ GGC ATC AGA CAC ATA TCA CTG 3′ 5′ TGT GGC ATT GAG GGT AGC 3′	UBC901	CACACACACACACACARY
11	Vm33	5′ GCA CGA GAT CTG GTG CTC CTT 3′ 5′ CAG CGA GCG CGA ACC 3′		
	
12	Vm39	5′ GAT GGT TGT AAT GGG AGA GTC 3′ 5′ AAA AGG ATG AAA TTA GGA GAG CA 3′		
	

#### 2.4.3 Agarose gel electrophoresis of PCR products

The agarose gel electrophoresis to visualize the PCR products were prepared according to Armstrong and Schulz (2008) with some modifications. Briefly, placing 1.5 mg agarose in 100 ml Tris‐Borate EDTA (TBE) buffer and then boiling it in a water bath. Ethidium bromide (5 µl) was added to the melted gel after the temperature decreased to 55°C. The melted gel was poured into a mini‐gel apparatus and the comb was immediately inserted, then the comb was removed when the gel hardened, and the gel was covered by TBE buffer. Eight μL of PCR products were loaded in each well and run at 80 V. PCR products were visualized through UV light using a UV spectrophotometer (Jenway, 6305) at 280 nm. The bands of SSR and ISSR‐PCR products were counted, scored, and ranked. The obtained bands were compared with the bands of the DNA ladder (Non‐Liner Dynamic Lth, USA).

All bright and visible fragments were scored as (1) if present or (0) if absent (Table S‐1,2). The banding patterns were studied taking into consideration the relative migration of their different sizes. To study the association between either SSR or ISSR‐PCR banding products and the cooking time of the studied cowpea genotypes, Spearman's rank correlation coefficient (Rs) was calculated according to Sokal and Rohlf (1995) and was determined using the following equation

$$\text{Rs}=1‐\left(\frac{6\Sigma d^{2}}{n^{3}‐n}\right)$$

Whereas *d*, the difference in ranks of the genotypes; *n*, the number of genotypes. The results were always between 1 (a perfect positive correlation) and −1 (a perfect negative correlation). The highest performance genotype presented the highest ranking value, and vice‐versa, for either cooking time or PCR banding products categories.

## 3 STATISTICAL ANALYSIS

An analysis of variance (ANOVA) test for the chemical composition and cooking treatment of cowpea genotypes was performed using CoStat software (version 6.4, Monterey, CA, USA). The obtained data were expressed as mean ± *SD*, and the differences were considered to be statistically significant for *p* ˂ .05.

## 4 RESULTS AND DISCUSSION

### 4.1 Chemical composition of cowpea seeds

The chemical composition of cowpea seeds seemed to be of great importance in their cooking quality. Table 2 shows the chemical composition of cowpea genotypes seeds before and after cooking. The data exhibited significant differences (*p* < .05) among cowpea genotypes for all the investigated chemical properties before and after cooking, except for the fiber content. Cowpea genotypes seeds presented different moisture content on the basis of their weights and/or sizes. The moisture contents ranged from 6.40% to 9.50% and 23.54% to 32.80% before and after cooking, respectively. The lowest values before and after cooking were recorded in Greenish Black Balady (6.40 ± 0.36 and 23.54 ± 0.62) while the highest values in Variegated Chinese (9.50 ± 0.28 and 32.80 ± 0.17) followed by Azmerlli (8.60 ± 0.16 and 29.34 ± 0.11), respectively. Cooked cowpea genotypes seeds showed an increase in moisture contents when compared to cowpea genotypes seeds before cooking. This increase can be explained by the imbibition of the cowpea seeds during cooking (Melo et al., 2017). Concerning the protein content, it ranged from 21.22% to 30.26% and 14.49% to 21.37% before and after cooking, respectively. Previous literature reported that cowpea seeds were rich in protein content, containing about 21%–31% proteins per 100 mg (Anam 2016; El‐Jasser, 2011; Henshaw & F.O., 2014), thus, they are considered a nutritionally balanced food for human. Moreover, previous studies declared that seeds of cowpea genotypes having high protein content could be selected for formulating infant feeds and for consumers characterized by protein‐deficiency conditions (Moses et al., 2018; Ravelombola et al., 2016). Black Balady genotype before cooking exhibited the highest content (30.26 ± 0.05) followed by Azmerlli (29.80 ± 0.08) and Greenish Black Balady (28.50 ± 0.00) genotypes. However, the genotypes of Buff Chinese (21.22 ± 0.10) and Kafr‐ElSheikh (21.92 ± 0.41) showed the lowest content. The results are in agreement with Hamid et al. (2015) who mentioned that protein content was higher in black cowpea seeds than the white or red seeds. Besides, cowpea genotypes seeds after the cooking process showed a reduction in protein when compared to uncooked cowpea genotypes seeds. This reduction in protein contents may be due to their loss during the cooking, as a small amount of amino acids may have been solubilized in the cooking water, causing a decrease in the protein content of the seeds (Ravelombola et al., 2016). Considering the content of the nonprotein substance (total carbohydrates, lipids, and vitamins) in cowpea seeds, a reverse trend was evidenced with respect to the protein contents. Cowpea genotypes exhibited low protein content with high nonprotein substances content, particularly in the case of Buff Chinese and Kafr‐ElSheikh genotypes. The results are in agreement with former studies that stated cowpea seeds are rich in carbohydrate content, which makes them an important source of energy for consumers (Ajeigbe et al., 2008; Ngoma et al., 2018). On the other hand, low carbohydrate genotypes were desirable in making meals for diabetic patients. The ash and fiber contents of the investigated cowpea genotypes seeds ranged from (3.34% to 4.13%), (2.26% to 3.05%) and (1.1% to 1.7%), (1.12% to 1.68%) before and after cooking, respectively. Regarding ash and fiber contents in cowpea seeds, the detected trend was similar to that of the moisture content. Greenish Black Balady manifested the lowest contents, whereas the Variegate Chinese and Azmerlli genotypes showed the highest contents. These findings comply with (Asante et al., 2020; El‐Jasser, 2011; Kamara et al., 2018; Silva et al., 2018). Cowpea genotypes seeds after cooking showed a reduction in ash when compared to uncooked cowpea genotypes seeds. This reduction in ash content can be attributed to the loss of minerals through diffusion in the water used in the thermal treatment (Melo et al., 2017). However, the cowpea genotypes seeds presented nearly similar fiber contents before and after the cooking process.

**TABLE 2:** Chemical composition (%) of studied cowpea genotypes before and after cooking*

Cowpea genotypes	Moisture		Protein		Non‐ protein		Ash		Fiber	
	Before	After	Before	After	Before	After	Before	After	Before	After
Azmerlli	8.60 ± 0.16 ^bB^	29.34 ± 0.11 ^bA^	29.80 ± 0.08 ^bA^	20.42 ± 0.40 ^bB^	55.87 ± 0.16 ^iA^	45.93 ± 0.56 ^iB^	4.13 ± 0.10 ^aA^	2.73 ± 0.04 ^bB^	1.60 ± 0.14 ^abA^	1.58 ± 0.11 ^abA^
Kareem 7	8.30 ± 0.16 ^bcB^	28.19 ± 0.21 ^cA^	27.62 ± 0.31 ^dA^	19.10 ± 0.43 ^dB^	59.24 ± 0.37 ^gA^	49.08 ± 0.68 ^hB^	3.54 ± 0.04 ^deA^	2.34 ± 0.02 ^cB^	1.30 ± 0.14 ^bcdA^	1.29 ± 0.09 ^deA^
Kafr‐ElSheikh	8.30 ± 0.22 ^bcB^	28.19 ± 0.00 ^cA^	21.92 ± 0.41 ^kA^	14.49 ± 0.46 ^iB^	64.50 ± 0.84 ^bA^	53.47 ± 0.62 ^cB^	3.88 ± 0.23 ^bA^	2.46 ± 0.17 ^cB^	1.40 ± 0.22 ^abcdA^	1.39 ± 0.60 ^cdA^
Doki 331	8.07 ± 0.09 ^cB^	27.42 ± 0.07 ^dA^	25.48 ± 0.31 ^fA^	17.12 ± 0.26 ^fB^	61.62 ± 0.34 ^dA^	52.31 ± 0.44 ^deB^	3.64 ± 0.00 ^cdeA^	2.40 ± 0.06 ^cB^	1.20 ± 0.08 ^cdA^	1.19 ± 0.13 ^efA^
Kahaa 1	8.20 ± 0.24 ^bcB^	27.81 ± 0.08 ^cdA^	26.30 ± 0.22 ^eA^	18.02 ± 0.02 ^eB^	60.45 ± 0.22 ^fA^	50.40 ± 0.16 ^gB^	3.75 ± 0.06 ^bcdA^	2.48 ± 0.04 ^cB^	1.30 ± 0.08 ^bcdA^	1.29 ± 0.06 ^deA^
White Balady	7.10 ± 0.16 ^dB^	24.30 ± 0.06 ^eA^	23.24 ± 0.17 ^iA^	15.89 ± 0.09 ^gB^	64.71 ± 0.32 ^bA^	56.05 ± 0.15 ^aB^	3.55 ± 0.00 ^deA^	2.34 ± 0.02 ^cB^	1.40 ± 0.16 ^abcdA^	1.42 ± 0.08 ^cdA^
Red Balady	6.50 ± 0.08 ^eB^	23.98 ± 0.26 ^eA^	27.29 ± 0.06 ^dA^	19.72 ± 0.07 ^cB^	61.34 ± 0.41 ^deA^	52.60 ± 0.22 ^dB^	3.47 ± 0.21 ^eA^	2.31 ± 0.07 ^cB^	1.40 ± 0.24 ^abcdA^	1.39 ± 0.01 ^cdA^
Black Balady	6.60 ± 0.16 ^eB^	24.42 ± 0.09 ^eA^	30.26 ± 0.05 ^aA^	21.30 ± 0.21 ^aB^	58.33 ± 0.18 ^hA^	50.50 ± 0.22 ^gB^	3.51 ± 0.03 ^eA^	2.31 ± 0.08 ^cB^	1.30 ± 0.00 ^bcdA^	1.29 ± 0.03 ^deA^
Greenish Black Balady	6.40 ± 0.36 ^eB^	23.54 ± 0.62 ^fA^	28.50 ± 0.00 ^cA^	21.37 ± 0.05 ^aB^	60.57 ± 0.53 ^efA^	51.71 ± 0.57 ^efB^	3.43 ± 0.10 ^eA^	2.26 ± 0.06 ^cB^	1.10 ± 0.08 ^dA^	1.12 ± 0.04 ^fA^
White Brazilian	8.50 ± 0.00 ^bcB^	28.98 ± 0.16 ^bA^	22.80 ± 0.08 ^jA^	15.15 ± 0.04 ^hB^	63.41 ± 0.24 ^cA^	51.88 ± 0.30 ^deB^	3.79 ± 0.06 ^bcA^	2.50 ± 0.16 ^bcB^	1.50 ± 0.22 ^abcA^	1.49 ± 0.08 ^bcA^
Black Chinese	8.10 ± 0.29 ^cB^	27.42 ± 0.04 ^dA^	24.99 ± 0.28 ^hA^	16.79 ± 0.05 ^fB^	61.92 ± 0.32 ^dA^	52.03 ± 0.09 ^deB^	3.59 ± 0.03 ^cde^	2.37 ± 0.04 ^c^	1.40 ± 0.08 ^abcdA^	1.39 ± 0.07 ^cdA^
Purple Chinese	8.30 ± 0.14 ^bcB^	28.19 ± 0.13 ^cA^	25.43 ± 0.15 ^fgA^	17.12 ± 0.07 ^fB^	61.33 ± 0.27 ^deA^	51.00 ± 0.27 ^fgB^	3.64 ± 0.02 ^cdeA^	2.40 ± 0.07 ^cB^	1.30 ± 0.00 ^bcdA^	1.29 ± 0.03 ^deA^
Buff Chinese	8.10 ± 0.14 ^cB^	27.42 ± 0.08 ^dA^	21.22 ± 0.10 ^iA^	14.49 ± 0.13 ^iB^	65.76 ± 0.18 ^aA^	54.41 ± 0.20 ^bB^	3.62 ± 0.02 ^cdeA^	2.39 ± 0.06 ^cB^	1.30 ± 0.14 ^bcdA^	1.29 ± 0.06 ^deA^
Variegate Chinese	9.50 ± 0.28 ^aB^	32.80 ± 0.17 ^aA^	25.02 ± 0.01 ^ghA^	16.82 ± 0.38 ^fB^	60.16 ± 0.21 ^fA^	45.84 ± 0.30 ^iB^	3.62 ± 0.06 ^cdeA^	3.05 ± 0.32 ^aA^	1.70 ± 0.22 ^aA^	1.68 ± 0.04 ^aA^

Note

Values presented as means of triplicate ±standard deviation.

Values with the different superscript letter (lowercase, different cowpea genotypes seeds, within a column) are significantly different (*p* < .05).

Values with the different superscript letter (uppercase, same genotype before and after, within a row) are significantly different (*p* < .05).

* Cooking process conditions; the seeds are soaked in tap water for 4 hr, then placed in 100 ml of tap water and cooked in a boiling water bath until the end of cooking.

### 4.2 Cooking time

Cooking time is considered the main indicator of cowpea seeds cooking quality and the pivotal factor in the choice of a particular variety by the consumers. Taking into account that the nutritional value of cowpea seeds strongly depends on their nutrients amounts and that the nutrients could be lost during the cooking process, any cooking treatment that efficiently reduces the cowpea seeds cooking time is highly beneficial (Hamid et al., 2015). The cooking times of cowpea seed genotypes estimated under different cooking treatments are compared in Table 3. The obtained results exhibited significant differences among all genotypes and cooking treatments, reflecting different reactions, and cooking time influence, correlated to their different genetic backgrounds. Similar results were previously reported about rapid methods to evaluate the cowpea cooking properties by soaking dry seeds for 12 hr, followed by cooking for 27 min (Mashi, 2006; Yeung et al., 2009). The authors found significant differences in cooking quality among the studied 52 cowpea cultivars (Mashi, 2006; Yeung et al., 2009).

**TABLE 3:** Cooking time (min) of studied cowpea genotypes under different cooking treatments

Cowpea genotypes	Unsoaked treatments		Soaked treatments				
	Dry seeds	Toasted seeds	Distilled water	Tap water	Sodium bicarbonate solution	Baking powder solution	Tap water and microwave
Azmerlli	64.00 ± 0.82 ^aA^	39.00 ± 2.94 ^bB^	20.00 ± 1.63 ^efC^	17.00 ± 0.82 ^deCD^	15.00 ± 0.82 ^cDE^	12.00 ± 0.82 ^cdE^	8.70 ± 0.22 ^cdF^
Kareem 7	23.00 ± 1.63 ^gA^	23.30 ± 0.36 ^hA^	21.00 ± 0.82 ^deB^	16.00 ± 0.00 ^efC^	13.00 ± 0.82 ^cdeD^	11.00 ± 0.82 ^cdE^	5.00 ± 0.82 ^ghF^
Kafr‐ElSheikh	45.00 ± 1.63 ^cA^	38.00 ± 0.82 ^bB^	21.00 ± 0.00 ^deC^	17.00 ± 1.63 ^deD^	13.00 ± 1.41 ^cdeE^	10.00 ± 0.82 ^deF^	7.00 ± 0.82 ^efG^
Doki 331	44.00 ± 2.16 ^cdA^	34.30 ± 0.00 ^cdB^	20.00 ± 1.63 ^efC^	19.00 ± 0.82 ^cdC^	15.00 ± 1.63 ^cD^	10.00 ± 1.63 ^deE^	6.00 ± 0.00 ^fgF^
Kahaa 1	40.52 ± 0.38 ^deA^	30.00 ± 4.08 ^efB^	17.00 ± 0.82 ^gC^	15.00 ± 0.82 ^efCD^	12.00 ± 0.82 ^deD^	8.00 ± 0.82 ^eE^	4.00 ± 0.82 ^hiF^
White Balady	47.70 ± 0.50 ^bcA^	28.00 ± 0.82 ^fB^	18.00 ± 0.00 ^fgC^	17.00 ± 1.63 ^deC^	12.67 ± 1.70 ^cdeD^	10.00 ± 1.42 ^deE^	4.00 ± 0.00 ^hiF^
Red Balady	48.00 ± 0.82 ^bcA^	27.00 ± 2.16 ^fgB^	17.00 ± 0.82 ^gC^	16.00 ± 0.82 ^efCD^	14.00 ± 0.82 ^cdDE^	12.00 ± 1.41 ^cdE^	5.30 ± 0.08 ^ghF^
Black Balady	47.00 ± 1.63 ^bcA^	30.00 ± 0.00 ^efB^	16.00 ± 1.41 ^gC^	15.00 ± 0.00 ^efCD^	13.00 ± 1.42 ^cdeDE^	11.00 ± 1.42 ^cdE^	5.70 ± 0.08 ^fgF^
Greenish Black Balady	32.70 ± 0.14 ^fA^	24.00 ± 0.82 ^ghB^	16.00 ± 0.00 ^gC^	14.00 ± 1.63 ^fC^	11.00 ± 0.82 ^eD^	8.00 ± 1.42 ^eE^	3.00 ± 0.82 ^iF^
White Brazilian	50.00 ± 4.08 ^bA^	32.00 ± 0.82 ^deB^	24.00 ± 0.82 ^cC^	21.00 ± 0.82 ^cC^	13.00 ± 0.00 ^cdeD^	13.00 ± 0.82 ^cD^	8.00 ± 0.82 ^deE^
Black Chinese	47.00 ± 2.16 ^bcA^	33.00 ± 0.82 ^cdeB^	28.00 ± 2.16 ^bC^	25.00 ± 1.63 ^bC^	19.00 ± 0.82 ^bD^	19.00 ± 0.82 ^bD^	9.70 ± 0.16 ^cE^
Purple Chinese	40.00 ± 4.08 ^deA^	33.00 ± 0.82 ^cdeB^	23.00 ± 1.63 ^cdC^	20.00 ± 0.00 ^cC^	13.00 ± 1.63 ^cdeD^	13.00 ± 1.42 ^cD^	8.00 ± 1.63 ^deE^
Buff Chinese	37.00 ± 1.41 ^eA^	36.00 ± 0.82 ^bcA^	30.00 ± 0.82 ^bB^	26.00 ± 2.16 ^bC^	18.00 ± 1.63 ^bD^	18.00 ± 0.82 ^bD^	12.00 ± 0.82 ^bE^
Variegate Chinese	61.70 ± 1.75 ^aA^	53.33 ± 0.47 ^aB^	34.00 ± 1.41 ^aC^	29.00 ± 0.82 ^aD^	26.00 ± 0.82 ^aE^	26.00 ± 0.82 ^aE^	20.00 ± 0.00 ^aF^

Note

Values presented as means of triplicate ± standard deviation.

Values with the different lowercase superscript letter within a column are significantly different (*p* < .05).

Values with the different uppercase superscript letter within a row are significantly different (*p* < .05).

Azmerlli and Variegated Chinese seeds, both unsoaked, air‐dried, and toasted seeds, presented a longer time to cook, probably due to their larger and rounder shape, followed by the white Brazilian. On the contrary, Kareem 7 achieved the shortest cooking time, followed by the Greenish Black Balady. The other genotypes were, similar in cooking time, more or less. The observed differences in cooking time between air‐dried and toasted seeds might be due to the effect of toasting which removed all free and bound waters from seeds shortening their cooking time. The cowpea seeds toasting treatment seemed to be a new approach for the improvement of the cowpea cooking quality. However, the soaking of cowpea seeds before cooking had good effects on cooking ability, making the seeds easier to digest. Overall, the cooking time under distilled water was longer than that under tap water. This might be due to the existence of some soluble salts in tap water which might improve cooking time and quality (Melo et al., 2017). In detail, the Variegated Chinese recorded the longest time, under both water soaking treatments, followed by Buff Chinese, whereas Greenish Black Balady and Black Balady achieved the shortest cooking time under both types of water soaking treatments. Similar results were obtained in some cowpea varieties from different African countries, differing in their agronomic attributes, for their cooking quality. These varieties exhibited genetic variations in cooking time with or without water soaking. Cooking time ranged from 29 to 64 min without soaking, and from 25 to 50 min after soaking, showing the smallest seed varieties the longest time (Liu et al., 2005; Wood, & Jennifer, 2016).

Concerning the soaked treatments of cowpea seeds in sodium bicarbonate or baking powder solutions, all the studied cowpea genotypes behaved in the same manner as distilled and tap water treatments. Variegated Chinese achieved the longest cooking time followed by Black Chinese, whereas Greenish Black Balady accomplished the shortest cooking time. The shorter cooking time in most of the cowpea genotype seeds under baking powder soaking solution compared to the treatment in sodium bicarbonate solution before cooking might be due to that baking powder composition: in fact, it contains further ingredients, such as sodium pyrophosphate and corn meal, in addition to sodium bicarbonate which could lead to improving the cooking time and quality of seeds (Bhokre & Joshi, 2015). Adding baking powder to soaking and cooking solutions of cowpea seeds seemed to be a new approach for improving the cowpea cooking quality. Regarding the exposure of soaked cowpea seed to microwave, Variegated Chinese presented the longest cooking time, followed by Buff Chinese, while Greenish Black Balady the shortest cooking time, followed by White Balady and Kahaa 1. Thus, it is possible to conclude that the exposure of cowpea seeds to microwaves before cooking appeared to be a promising approach in cowpea cooking to improve their quality.

Generally, cowpea seeds consumption might be limited owing to the presence of several anti‐nutrient factors such as tannins, phytate, and trypsin inhibitors, which reduced their availability (Khalid & Elhardallou, 2016). Soaking process in water before cooking might remove such factors: during soaking, cowpea seeds undergo physiochemical changes leading to softening the seed tissues and hence promoting a shorter cooking time (Hamid et al., 2016; Kouam et al., 2018; Yeung et al., 2009). Moreover, the soaking of cowpea seeds prior to cooking would remove the seed coat pigmentation and polyphenols; also, the content of proteins, sugars, and ash would be affected, which diffuse in the soaking solution. Finally, the soaking of cowpea seeds before cooking would reduce the risk of flatulence due to the oligosaccharides, which can be fermented by bacteria‐producing gas. For these reasons, Hawaiian cooks usually add few amounts of ginger to the cooking solutions in order to decrease the potential development of gases (Animasaun et al., 2015; Ngalamu et al., 2015; Yeung et al., 2009).

Finally, the ranking pattern of the studied cowpea genotypes according to their estimated cooking under different cooking treatments for genotypic evaluation is presented in Table 4. The highest score (14) was revealed in the case of the genotype with the shortest cooking time, whereas the lowest score (1) in the case of longest cooking time as expected. Then, the mean rank of each genotype was estimated. The data showed that Kareem 7 ranked first with the shortest cooking time, followed by the Greenish Black Balady and Kahaa 1. On the other hand, the Variegate Chinese genotype ranked last with the longest cooking time, followed by Buff Chinese and Black Chinese. Thus, on the basis of all these collected results, it is worthily noting that it is important to choose cowpea genotypes having a shorter cooking time, in order to improve the cowpea cooking quality (Beshir et al., 2019).

**TABLE 4:** Ranking pattern of studied cowpea genotypes according to their cooking time under different treatments

Cowpea genotypes	Unsoaked treatments		Soaked treatments					
	Dry seeds	Toasted seeds	Distilled water	Tap water	Sodium bicarbonate solution	Baking powder solution	Tap water and microwave	Mean ranks of cooking time
Azmerlli	1	2	6	9	6	7	6	5.28
Kareem 7	13	14	8	14	11	14	14	12.57
Kafr‐ElSheikh	8	3	8	9	11	13	7	8.43
Doki 331	9	5	9	6	6	13	8	8.00
Kahaa 1	10	10	12	13	13	8	13	11.28
White Balady	5	11	10	9	13	13	13	10.57
Red Balady	4	12	12	11	4	7	10	8.57
Black Balady	7	10	14	13	11	8	9	10.28
Greenish Black Balady	14	13	14	11	11	13	11	12.42
White Brazilian	3	8	4	4	11	8	5	6.14
Black Chinese	7	7	6	3	2	3	3	4.42
Purple Chinese	11	7	5	5	11	13	5	8.14
Buff Chinese	12	4	2	2	3	2	1	3.71
Variegate Chinese	2	1	1	1	1	1	2	1.28

### 4.3 Molecular studies

**FIGURE 2:**
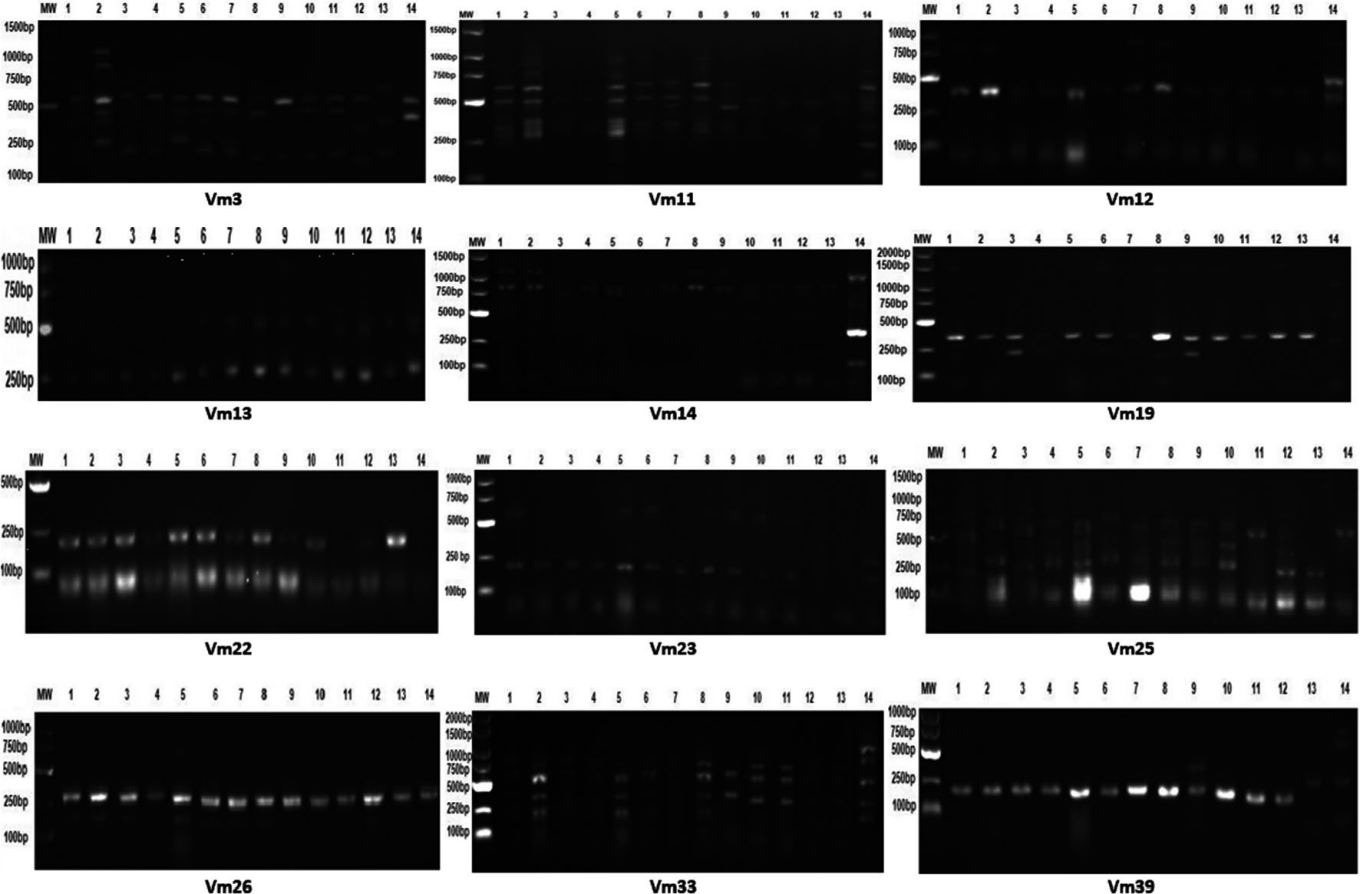
Electrophoretic banding patterns of SSR primers produced from the studied cowpea genotypes

**FIGURE 3:**
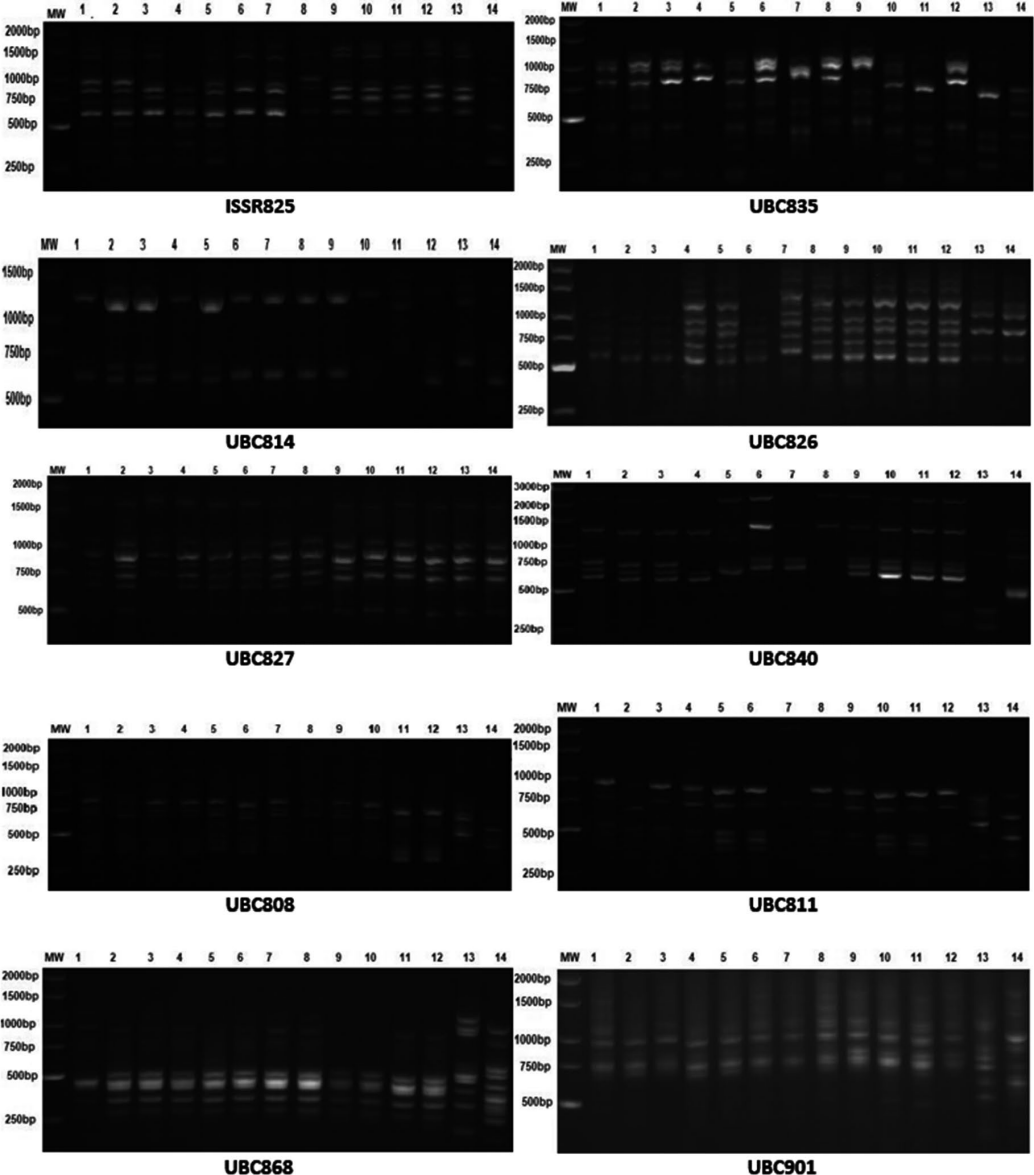
Electrophoretic banding patterns of ISSR primers produced from the studied cowpea genotypes

Molecular markers appeared to be useful tools for assessing genetic variations at the molecular level (Araújo et al., 2019). The electrophoretic banding patterns of SSR and ISSR primers produced from cowpea genotypes are illustrated in Figures 2 and 3, respectively. The distribution of SSR and ISSR bands among cowpea genotypes is given in Table 5. The twelve SSR primers amplified 447 bands and were widely and differently distributed among cowpea genotypes. In this respect, Maithreyee (2011) characterized 32 cowpea cultivars using 20 SSR primers, whereas Buels et al. (2016) announced that 500 SSR primers yielded 54 bands among 32 cowpea accessions. On the other hand, the ten ISSR primers amplified a total number of 892 bands widely distributed among genotypes. Similar behaviors were reported in studies about the genetic diversity among some cowpea cultivars and accessions using SSR and ISSR markers, successfully designing many primer pairs (Anatala et al., 2014; Badiane et al., 2012). The data revealed that the number of detected SSR bands among cowpea genotypes was lower than those of ISSR primers, indicating that the action of SSR primers is different from the ISSR primers one. Moreover, Araújo et al. (2019) investigated some Brazilian cowpea landraces using 25 ISSR primers and stated that only 14 primers amplified 80 bands.

**TABLE 5:** Distribution and ranking pattern of the PCR bands of simple sequence repeat (SSR), intersimple sequence repeat (ISSR) primers, and cooking time among studied cowpea genotypes

Cowpea genotypes	SSR		ISSR		Mean ranks of cooking time
	Distribution bands	Ranks	Distribution bands	Ranks	
Azmerlli	38	11	62	3	5.28
Kareem 7	36	10	63	4	12.57
Kafr‐ElSheikh	34	8	66	6	8.43
Doki 331	35	9	60	1	8.00
Kahaa 1	35	9	65	5	11.28
White Balady	33	7	66	6	10.57
Red Balady	28	3	61	2	8.57
Black Balady	30	5	61	2	10.71
Greenish Black Balady	31	6	65	5	12.42
White Brazilian	28	3	66	6	6.14
Black Chinese	25	1	61	2	4.42
Purple Chinese	29	4	66	6	8.14
Buff Chinese	27	2	67	8	3.71
Variegate Chinese	38	11	63	4	1.28
Total bands	477		892		

Data in Table 5 also show the ranking patterns of SSR and ISSR‐PCR products, banding profiles, and ranking of seeds cooking time of the studied cowpea genotypes. The larger value gave the larger rank score for SSR or ISSR‐PCR products. These ranking patterns were subjected to the Spearman's rank correlations with the aim to detect any association between SSR or ISSR banding profile and the cooking time. Data revealed that the calculated Spearman's rank correlations (Rs) values with cooking time were 0.327 and 0.112 for SSR and ISSR bands, respectively. These estimates were smaller than the tabulated value of 0.05 which was 0.538, reflecting the existence of rank correlations between SSR or ISSR‐PCR products and the cooking time of cowpea seeds. It was difficult to compare our results with the previous studies because there are no such studies that linked cooking time in cowpea to molecular markers.

## 5 CONCLUSION

The cooking time is considered the main indicator of the cooking quality of cowpea seeds and an important factor in the selection of a particular variety by the consumers. The reduction in cooking time seems to be advantageous, requiring less energy and fuel, and mainly reducing the possible loss of cowpea seeds nutrients, thus improving their nutritional value. Among the fourteen investigated cowpea seed genotypes, the Egyptian cultivar Kareem 7 achieved the shortest cooking time, and the Black Balady genotypes manifested the greatest protein amount in their cooked seeds with respect to the other ones. Concerning the investigation of the genetic variations at the molecular level, the twelve SSR primers amplified 447 bands and were widely and differently distributed among the cowpea genotypes. In comparison, the ten ISSR primers amplified a total number of 892 bands, widely distributed among the studied genotypes. The association between the mean cooking times and SSR or ISSR‐PCR banding products of the studied cowpea genotypes was estimated in underserved trials. Such a study appeared to be a new approach and, for the first time, was performed in Egypt. Ultimately, the use of SSR and ISSR primers gave a clearer picture as effective tools in assessing and detecting the molecular polymorphisms existing among the studied cowpea genotypes and assuring their phylogenetic relationships.

## CONFLICT OF INTEREST

The authors declare that they do not have any conflict of interest.

## AUTHOR CONTRIBUTIONS

**Salma A. Soaud:** Conceptualization (equal); formal analysis (equal); methodology (equal); writing‐original draft (equal). **Salama M. Abdel‐Sayyed:** Supervision (equal); writing–review and editing (equal). **Elsayed M. I. Mahgoub:** Supervision (equal); writing–review and editing (equal). **Sameh A. Korma:** Formal analysis (equal); investigation (equal); writing–review and editing (equal). **Ilaria Cacciotti:** Writing–review and editing (equal). **Abdelmoneim H. Ali:** Writing–review and editing (equal). **Azhari Siddeeg:** Resources (equal). **Hany A. M. Wafa:** Supervision (equal); writing–review and editing (equal).

## ETHICAL APPROVAL

This study does not involve any human or animal testing.

## Data Availability

The dataset supporting the conclusions of this article is included within the manuscript.
